# Association of the rs688 Polymorphism in the Gene Encoding Low‐Density Lipoprotein Receptor in Bangladeshi Population With Coronary Artery Disease

**DOI:** 10.1155/genr/5670428

**Published:** 2026-04-09

**Authors:** Imran Hossain, Nahid Sharmin, Istiaque Ahmed, Golam Saklayen, Sauda Sumaya Dina, Sheikh Zahir Raihan

**Affiliations:** ^1^ Department of Clinical Pharmacy and Pharmacology, Faculty of Pharmacy, University of Dhaka, Dhaka, 1000, Bangladesh, du.ac.bd; ^2^ Department of Pharmaceutical Technology, Faculty of Pharmacy, University of Dhaka, Dhaka, 1000, Bangladesh, du.ac.bd; ^3^ Cardiac Surgery Department, Ibrahim Cardiac Hospital and Research Institute, Dhaka, 1000, Bangladesh

**Keywords:** coronary artery disease, genotype frequency, LDLR rs688, lipid profile, single-nucleotide polymorphism

## Abstract

**Background:**

Coronary artery disease (CAD) is among the leading causes behind the morbidity and mortality of the world population. Among others, low‐density lipoprotein (LDL) is one of the main drivers behind the development of CAD, and the LDL receptor (LDLR) plays a central role in cholesterol homeostasis by facilitating the clearance of LDL cholesterol from the blood. The LDLR rs688 single‐nucleotide polymorphism in exon 12 has been reported to influence mRNA splicing efficiency, potentially modifying receptor function and lipid metabolism. The aim of this study was to assess the association between LDLR rs688 polymorphism and CAD susceptibility among CAD patients in the Bangladeshi population and to determine its relationship with serum LDL level.

**Methods:**

A case–control study was conducted involving 225 participants, including 150 CAD patients and 75 healthy controls. High‐density lipoprotein, LDL, triglycerides, and total cholesterol were measured by biochemical tests using appropriate kits. The LDLR genotype was identified using the allele‐specific PCR (AS‐PCR) technique.

**Results:**

Relative to the control group, the CAD group showed a higher distribution frequency of the TT genotype (17.33%) and a lower frequency of the CC genotype (15.33%). The LDLR rs688 TT genotype showed significant association with CAD among Bangladeshi patients (OR = 3.617, 95% CI: 1.089–10.05; *p* = 0.0352). Furthermore, individuals carrying the TT and CT genotypes exhibited higher LDL levels compared with those carrying the CC genotype (*p* < 0.05). Finally, univariate and multivariate logistic regression analyses revealed that the LDLR rs688 TT genotype remained significantly associated with CAD after adjustments for covariates (*p* < 0.05).

**Conclusion:**

This hospital‐based case–control study provides preliminary evidence of an association between the LDLR rs688 TT genotype and CAD in a Bangladeshi population. These findings are preliminary and require validation in larger, population‐based studies.

## 1. Introduction

Coronary artery disease (CAD), the most prevalent form of heart disease, contributes significantly to the global morbidity and mortality across both sexes, irrespective of the racial and ethnic background [1, 2]. CAD has multifactorial etiology, resulting from a complex interaction of genetic predispositions and environmental influences. Well‐recognized risk factors, including dyslipidemia, diabetes mellitus, hypertension, and obesity, play a central role in the onset and progression of CAD, which is fundamentally driven by the underlying atherosclerosis process [3]. Moreover, epidemiological and genetic investigations have shown that specific gene polymorphisms are associated with an increased susceptibility to CAD across varying risk groups. Exploring these genetic determinants may enhance risk assessments and contribute to improved strategies for CAD prevention and management.

The low‐density lipoprotein receptor (LDLR) is a cell surface glycoprotein that binds and internalizes plasma low‐density lipoprotein (LDL) particles, playing a key role in maintaining cellular cholesterol homeostasis [4]. Polymorphic variants of the LDLR gene have been shown to markedly elevate plasma LDL concentrations, thereby contributing to an increased risk of atherosclerosis and CAD [5]. Numerous LDLR mutations have been identified affecting exonic regions, splice sites, and promoter sequences. Certain of these have been implicated in the pathogenesis of hypercholesterolemia [6, 7].

The single‐nucleotide polymorphism (SNP) rs688, positioned within exon 12 of the LDLR gene, has been identified as a genetic variant associated with alterations in LDL levels and susceptibility to CAD, irrespective of gender [8]. Notably, the TT genotype of rs688 has demonstrated significant associations with hyperlipidemia and CAD [9], along with elevated total and LDL cholesterol levels [10, 11]. Besides, the T allele of rs688 has shown a significant association with dyslipidemia and increased cardiovascular disease (CVD) risk [12]. This synonymous SNP interferes with the splicing enhancer element, leading to alternative exon splicing that may cause a frameshift and subsequently alter the resulting gene transcript [10].

It is important to note that allele frequencies and their associations can differ significantly among various ethnic populations, including European Caucasians, Africans, Americans, Asians, and Hispanics. Consequently, genetic associations identified in one population may not necessarily be applicable to others [13]. In this study, the rs688 polymorphism in the LDLR gene was selected as a candidate variant because previous studies have suggested its functional relevance in lipid metabolism and CVD susceptibility. This synonymous SNP, located in exon 12 of the LDLR gene, has been reported to influence mRNA splicing efficiency and LDL receptor function, potentially altering circulating LDL cholesterol levels and contributing to the development of CAD [10, 12]. However, the distribution and disease association of this polymorphism may vary across populations. Therefore, the findings from previously published studies cannot be directly generalized to the Bangladeshi population. Moreover, to date, no research has investigated the association of the rs688 SNP with susceptibility to CAD in the Bangladeshi population. Accordingly, the present study was undertaken to evaluate the frequency of LDLR (rs688) gene polymorphism and its potential relationship with CAD in the Bangladeshi population.

## 2. Materials and Methods

### 2.1. General Information

A hospital‐based case–control design was employed in this study. From May 2025 to October 2025, we recruited 150 Bangladeshi patients who were admitted and underwent angiography at Ibrahim Cardiac Hospital and Research Institute, Dhaka, Bangladesh, and were identified as confirmed CAD cases by expert physicians. Inclusion criteria were CAD patients aged ≥ 18 years without any congenital cardiovascular problems. Patients who were pregnant or had a history of stroke, hepatic or renal failure, severe infection, tumors, or severe heart failure (LVEF < 35%) were excluded from the study. A total of 75 angiography‐confirmed healthy participants were recruited from different tertiary‐level hospitals in Bangladesh as a control group. These individuals underwent coronary angiography due to suspected CAD but were subsequently found to have normal coronary arteries and no angiographic evidence of CAD. Accordingly, healthy controls were individuals without clinical or angiographic evidence of CAD and with no prior history of myocardial infarction, coronary revascularization, or other diagnosed CVD. The sample size was estimated using G∗Power software (Version 3.1.9.7) based on genotype frequency differences reported in a previous study investigating the association between the LDLR rs688 polymorphism and CVD [14]. For a two‐sided test, with *α* = 0.05, power = 90%, and a control‐to‐case ratio of 0.50, the required sample size was estimated to be 160 participants (107 cases and 53 controls). The present study included 225 participants (150 cases and 75 controls), exceeding the minimum requirement. Informed written consent was obtained from all participants, including both CAD patients and healthy controls. Subsequently, all the participants were interviewed using a structured questionnaire form to gather epidemiological, demographic, and medical information. Most participants were urban residents from the city centers of major Bangladeshi cities (e.g., Dhaka, Chattogram, Rangpur, Barisal, and Sylhet), with the remainder residing in adjacent rural areas. All participants self‐identified as ethnic Bengali, which represents the predominant ethnic group in Bangladesh, thereby reducing the likelihood of substantial population stratification in the study cohort. Patients who self‐reported receiving lipid‐lowering therapy at the time of sample collection were excluded from the study. However, reliable information on previous lipid‐lowering therapy could not be obtained for most of the patients, particularly those from the rural areas, due to recall bias and literacy limitations. Comprehensive laboratory and clinical data were also obtained from the study participants. This study protocol was approved by the Ethical Review Committee of the Faculty of Pharmacy, University of Dhaka, Dhaka‐1000 (Ref. No. Fa. Ph. E/053/2025).

### 2.2. Biochemical Analysis

Blood samples were collected from each participant using two different tubes: one containing an anticoagulant (EDTA) for genomic DNA extraction and the other without anticoagulant for serum separation and subsequent biochemical analysis. Total cholesterol (TC), triglycerides (TG), high‐density lipoprotein (HDL), and LDL levels were measured for each participant using commercially available enzymatic kits.

### 2.3. Genotyping

Genomic DNA was extracted from blood samples using the TIANamp Blood kit (TIANGEN, Beijing, China). The concentration and the purity of the extracted DNA were measured using the EzDrop 1000 Micro‐Volume Spectrophotometer (Blue‐Ray Biotech, Taipei, Taiwan). Genomic DNA was stored at −20°C. The rs688 polymorphism in the LDLR gene was identified by amplifying a 191 bp DNA fragment using an allele‐specific polymerase chain reaction (AS‐PCR). For each DNA sample, two separate PCR reactions were performed: one using the F‐Wild primer (5′‐CAC​TCC​ATC​TCA​AGC​ATC​GAT​GTC​AAC‐3′) and the other using the F‐Mutant primer (5′‐CAC​TCC​ATC​TCA​AGC​ATC​GAT​GTC​AAT‐3′), each paired with a common reverse primer (5′‐CAA​CCA​GTT​TTC​TGC​GTT​CAT​CTT​G‐3′). The primer design was adopted from Jha et al. [9]. To ensure DNA quality and PCR performance, each reaction also included an internal positive control primer pair (forward: 5′‐ACC​ACA​CCA​TCA​CCA​TCG‐3′ and reverse: 5′‐CCA​ACT​CAA​GTC​CAC​AGC‐3′), which consistently generated a 401 bp control band in all samples. The PCR reaction was performed in a total volume of 10 μL, containing 50 ng of genomic DNA, 5 μL of EmeraldAmp GT PCR Master Mix (Takara Bio Inc., Kusatsu, Japan), 0.2 μL of allele‐specific forward primer, 0.2 μL of common reverse primer, 0.5 μL positive‐control forward primer, 0.5 μL positive‐control reverse primer, and 3.1 μL of nuclease‐free water. PCR conditions were optimized, and finalized run conditions were as follows: an initial denaturation at 94°C for 3 min, followed by 35 cycles consisting of denaturation at 94°C for 30 s, annealing at 67°C for 30 s, and extension at 72°C for 1 min with a final elongation step at 72°C for 10 min. Amplified PCR products were subsequently stored at 4°C until further analysis. The resulting amplification products were visualized by gel electrophoresis on a 2% gel containing ethidium bromide.

Genotyping was performed in a manner blinded to the case and control status of the samples. To ensure accuracy, genotyping validation was conducted by amplifying the rs688 region with a different set of primers and performing sequencing on 10% of the randomly selected samples.

### 2.4. Statistical Analysis

Statistical analyses were performed using IBM SPSS Statistics Version 26.0 (IBM Corp., Armonk, NY, USA) and GraphPad Prism Version 10.3.0 (GraphPad Software, Boston, MA). Continuous variables were measured as mean ± standard deviation (SD), whereas categorical variables were summarized as frequencies and percentages. The independent samples *t*‐test was applied to compare continuous variables with a normal distribution, while the Mann–Whitney *U* test was applied for non‐normally distributed data. Differences between categorical variables were evaluated using the chi‐square or Fisher’s exact test where appropriate. Hardy–Weinberg equilibrium (HWE) for genotype distributions was tested by the chi‐square goodness‐of‐fit test with one degree of freedom. Multivariate logistic regression analysis was performed to identify the independent risk factors associated with CAD. A *p* value of < 0.05 was considered statistically significant.

## 3. Results

### 3.1. Comparison of Demographic and Anthropometric Data Between the CAD and the Control Groups

Clinical and demographic data were obtained from a total of 225 participants comprising 150 cases and 75 healthy controls. The baseline characteristics of the study population are summarized in Table 1. No statistically significant differences were observed in the measured demographic and anthropometric variables between the CAD and control groups.

**TABLE 1:** Demographic and anthropometric data of CAD and control groups.

Variables	CAD group	Healthy control group	*p* value
Age (years)	48.84 ± 12.98	45 ± 16.48	0.0579
Height (m)	1.59 ± 0.10	1.59 ± 0.08	0.9669
Weight (kg)	63.69 ± 12.88	65.22 ± 10.38	0.3716
BMI (kg/m^2^)	25.15 ± 3.13	25.00 ± 3.10	0.8070
Gender *n* (%)			0.2054
Male	114 (76.00%)	51 (68.00%)	
Female	36 (24.00%)	24 (32.00%)	
Smoking status *n* (%)			0.8221
Smoker	36 (24.00%)	16 (21.33%)	
Nonsmoker	94 (62.67%)	47 (62.67%)	
Past smoker	20 (13.33%)	12 (16.00%)	
Alcohol consumption *n* (%)			0.3333
Yes	0 (0.00%)	1 (1.33%)	
No	150 (100%)	74 (98.67%)	
Residence *n* (%)			0.6207
Urban	113 (75.33%)	59 (78.66%)	
Rural	37 (24.67%)	16 (21.33%)	
Diabetes *n* (%)			> 0.9999
Yes	68 (45.33%)	34 (45.33%)	
No	82 (54.67%)	41 (54.67%)	
Hypertension *n* (%)			0.2604
Yes	86 (57.33%)	37 (49.33%)	
No	64 (42.67%)	38 (50.67%)	

*Note:* Data expressed as *n* (%) and mean ± SD (standard deviation) where appropriate. *p* value was determined using the unpaired *t*‐test for continuous variables and chi‐square or Fisher’s exact test for categorical variables. *p* < 0.05 was considered as significant in comparison to control.

### 3.2. Comparison of Biochemical Parameters Between the CAD and the Control Groups

The biochemical parameters of the study participants are summarized in Table 2. The mean levels of TC and HDL cholesterol differed significantly between the CAD and control groups (*p* = 0.0043 and *p* = 0.0051, respectively). In contrast, TG and LDL values showed no statistically significant difference between the two groups (*p* > 0.05).

**TABLE 2:** Analysis of biochemical parameters of CAD and control groups.

Variables	CAD group	Healthy control group	*p* value
TC (mg/dL)	134.52 ± 34.14	148.49 ± 34.06	**0.0043^∗∗^**
TG (mg/dL)	157.46 ± 108.99	141.49 ± 71.55	0.1900
HDL (mg/dL)	30.85 ± 10.68	37.39 ± 18.28	**0.0051^∗∗^**
LDL (mg/dL)	76.03 ± 27.23	78.14 ± 39.95	0.6806

*Note:* Data expressed as mean ± SD (standard deviation). *p* value was determined by *t*‐test. Differences were considered significant compared with the control group at *p* < 0.05 (^∗^) and highly significant at *p* < 0.01 (^∗∗^) (marked in bold).

Further analysis demonstrated that participants carrying the LDLR rs688 CC genotype had significantly lower LDL‐C levels compared with those with the CT and TT genotypes, regardless of whether they belonged to the CAD or control group (*p* < 0.05) (Table 3).

**TABLE 3:** Comparison of serum LDL‐C level according to LDLR rs688 genotype irrespective of their case–control status.

Genotype	LDL cholesterol (mg/dL)
CC	62.18 ± 26.77
CT	79.46 ± 31.30^a^
TT	81.43 ± 36.78^b^

*Note:* Data expressed as mean ± standard deviation (SD). Comparisons performed using independent samples *t*‐test.

^a^Comparison between CC and CT groups, *p* < 0.001 (actual *p* = 0.0009).

^b^Comparison between CC and TT groups, *p* = 0.018.

### 3.3. LDLR rs688 Genotypic Distribution

The distribution of LDLR rs688 genotype among study participants is presented in Table 4.

**TABLE 4:** Genotypic and allelic frequencies of LDLR rs688 and carriage rates in healthy control and CAD groups.

Genotype frequency	CAD group		Healthy control group	
	Number	Frequency	Number	Frequency
CC	23	15.33%	16	21.33%
CT	101	67.33%	54	72.00%
TT	26	17.33%	5	6.67%

*Allele frequency*				
C	147	49.00%	86	57.33%
T	153	51.00%	64	42.67%

*Carriage rate*				
C (+)	124	82.67%	70	93.33%
C (−)	26	17.33%	5	6.67%
T (+)	127	84.67%	59	78.67%
T (−)	23	15.33%	16	21.33%

*Note:* Hardy–Weinberg equilibrium: *χ*2 = 16.68, *p* < 0.001 for control group.

**FIGURE 1:**
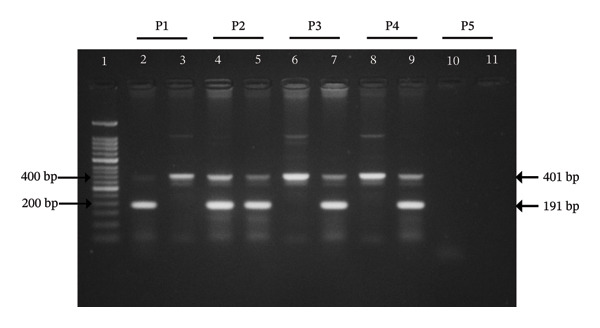
Allele‐specific PCR (AS‐PCR) analysis of the LDLR rs688 (C/T) polymorphism. A 191‐bp DNA fragment represents the presence of the corresponding allele, whereas the absence of amplification indicates the lack of that allele in the sample. A band at 401‐bp serves as a positive control. Lane 1 contains the 50 bp DNA ladder, and lanes 2–11 represent individual samples. Samples were run in pairs (P1–P5) with wild‐type and mutant‐specific forward primers. Each run also included the positive‐control forward and reverse primers which served as an indicator of DNA quality by producing a 401 bp band in all samples. Among the sample pairs, P1 denotes the homozygous CC genotype, P2 denotes the heterozygous CT genotype, P3 and P4 denote homozygous TT genotype, and P5 serves as the negative control. The PCR products were resolved on a 2% (w/v) agarose gel.

Both rs688C and rs688T alleles yielded PCR fragments of 191 bp as illustrated in Figure 1.

In the CAD group, individuals carrying the CC, CT, and TT genotypes were 23, 101, and 26, respectively, whereas in the control group, the corresponding frequencies were 16, 54, and 5. Statistical analysis revealed that both the CC vs TT comparison in the codominant model (OR = 3.617, 95% CI: 1.089–10.05, *p* = 0.0352) and the TT vs. CC + CT comparison in the recessive model (OR = 2.935, 95% CI: 1.068–7.273, *p* = 0.0387) demonstrated statistically significant associations with CAD (Table 5). No significant associations were observed under the dominant or allelic model. These findings suggest that the TT genotype of LDLR rs688 may be associated with increased susceptibility to CAD in the studied population.

**TABLE 5:** Distribution of the LDLR rs688 polymorphism according to the model of inheritance.

Genotype		Odds ratio	95% CI	*p* value
Codominant	CC vs TT	3.617	1.089–10.05	**0.0352** ^**∗**^
	CT vs TT	2.780	1.067–6.947	0.0558
	CC vs CT	1.301	0.6368–2.688	0.5761

Dominant	CT + TT vs CC	1.497	0.7458–3.031	0.2682

Recessive	TT vs CC + CT	2.935	1.068–7.273	**0.0387** ^**∗**^

Allele	C vs T	1.399	0.9356–2.069	0.1094

Carriage rate	C (+) vs C (−)	2.935	1.068–7.273	**0.0387** ^**∗**^
	T (+) vs T (−)	1.663	0.8390–3.316	0.1886

*Note:* The *p* values were calculated using the appropriate contingency table tests. A *p* value < 0.05 was considered statistically significant when compared to the control group. The bold values indicate statistically significant results (*p* < 0.05). Statistically significant differences between groups are indicated by asterisks: ^∗∗^ *p* < 0.01, ^∗^ *p* < 0.05.

Abbreviation: CI = confidence interval.

### 3.4. Univariate and Multivariate Regression Analyses for Risk Factors of CAD

Both univariate and multivariate logistic regression analyses were performed to find out the factors independently associated with CAD (Table 6). In the multivariate model, which is adjusted for age, gender, smoking status, hypertension, diabetes, and BMI, the LDLR rs688 TT genotype remained independently associated with CAD (OR = 3.568, 95% CI = 1.089–11.692, *p* = 0.036). None of the other covariates, including traditional risk factors, showed a significant association with CAD (all *p* > 0.05).

**TABLE 6:** Univariate and multivariate regression analyses for risk factors of CAD.

Variable	Univariate			Multivariate		
	OR	95% CI	**p** value	OR	95% CI	**p** value
Age	1.019	0.999–1.039	0.059	1.025	0.997–1.054	0.086
Gender	0.671	0.364–1.239	0.202	0.829	0.423–1.627	0.586
Smoking	0.859	0.441–1.674	0.655	0.835	0.399–1.745	0.631
Hypertension	0.725	0.415–1.264	0.257	0.764	0.339–1.719	0.515
Diabetes	1	0.573–1.745	1	2.04	0.906–4.591	0.085
BMI	1.018	0.932–1.113	0.687	1.037	0.945–1.137	0.447
Genotype CC	1			1		
Genotype CT	1.301	0.634–2.669	0.473	1.211	0.577–2.54	0.612
Genotype TT	3.617	1.145–11.428	**0.028** ^**∗**^	3.568	1.089–11.692	**0.036** ^**∗**^

*Note:* The bold values indicate statistically significant results (*p* < 0.05). Statistically significant differences between groups are indicated by asterisks: ^∗^ *p* < 0.05.

Abbreviations: CI = confidence interval, OR = odds ratio.

In the univariate analysis, the TT genotype was also significantly associated with CAD (OR = 3.617, 95% CI = 1.145–11.428, *p* = 0.028), whereas the CT genotype and other clinical parameters did not demonstrate significant effects.

## 4. Discussion

Bangladesh is undergoing an epidemiological transition, shifting from a predominance of communicable diseases to noncommunicable diseases (NCDs). Mortality attributable to chronic conditions, including CAD, is rising at an alarming pace [15]. The precise prevalence of CAD among the Bangladeshi population remains unclear [16]. Evidence from a rural cohort indicates a striking escalation in CVD between 1986 and 2006, with age‐standardized mortality rates increasing approximately 30‐fold among men (from 16 to 483 deaths per 100,000) and 47‐fold among women (from 7 to 330 deaths per 100,000) [17].

Traditional risk factors, including high‐fat diet, advanced age, smoking, hypertension, diabetes mellitus, and dyslipidemia, are well‐established contributors to an increased risk of CAD. The rs688 SNP, located near exon 12 of the LDLR gene, which encodes a receptor for the ApoE protein, has been shown to cause reduced mRNA splicing efficiency in males. Furthermore, recent genome‐wide association studies (GWAS) have revealed that common variants in the LDLR locus are strongly linked to a proatherogenic lipid profile and an elevated susceptibility to CAD [18].

In this study, we investigated the link between the LDLR rs688 polymorphism and CAD risk among individuals from Bangladesh. To our knowledge, this is the first study to examine the relationship between the LDLR rs688 and CAD risk in this specific population. Our findings demonstrated that the TT genotype was significantly more prevalent, while the CC genotype was less frequent in the CAD patients compared to the control group. Moreover, individuals carrying the CT and TT genotypes exhibited notably higher LDL‐C levels than those of the CC genotype, which is in line with a previous study where the CT + TT genotypes had significantly higher LDL levels compared to the CC genotype [19]. The results suggest that LDLR rs688 polymorphism may contribute to the susceptibility to CAD, with the TT genotype showing independent association in this cohort.

In our study, no significant difference in serum LDL‐C levels was observed between the CAD patients and the control group, which is in line with the findings of Jha et al. [9]. This lack of difference may be attributed to the complex and multifactorial nature of CAD, the reliance on a single cross‐sectional lipid measurement in capturing long‐term lipid exposure, and the potential influence of prior lipid‐lowering therapy that could not be fully ascertained despite exclusion of current statin users. However, genotype‐based analysis revealed that individuals carrying the CT and TT genotypes had higher LDL‐C levels compared with those carrying the CC genotype, regardless of case–control status. This suggests that the effect of the LDLR rs688 polymorphism on lipid metabolism may be better captured when individuals are stratified by genotype rather than by disease status alone. A higher frequency of the TT genotype (17.33%) was observed among CAD patients compared to healthy controls (6.67%) in our study. Similar trends were reported in studies from India (21% vs. 9%) [9], Italy (21.5% vs. 20.2%) [8], and the United States of America (21.4% vs. 14.5%) [20]. In contrast, studies conducted in Taiwan reported a lower prevalence of the TT genotype, with frequencies of 2% vs. 4% [21] and 3.2% vs. 3.95% [22] in CAD and control groups, respectively.

In line with the previous reports, our findings showed that the number of male participants was more than that of the female participants in both the CAD and the control groups, although differences were not statistically significant (Table 1). Similarly, the prevalence of hypertension and diabetes did not differ significantly between the CAD and the control groups. Moreover, age, smoking status, and BMI were not identified as significant risk factors for CAD in our study (all *p* > 0.05) (Table 6). This may reflect the comparable distribution of these variables between cases and controls as well as the relatively limited variability of these factors within the study cohort, which may have reduced their apparent effect in the multivariate regression model. Only HDL and TC levels differed significantly between the two groups, whereas LDL levels did not. The relatively lower TC observed in CAD patients may reflect prior lifestyle modifications, dietary changes, or earlier medical management before recruitment, which may influence lipid profiles measured during hospitalization. In addition, lipid measurements obtained at a single time point may not accurately reflect long‐term lipid exposure prior to disease onset.

Mutation in the LDLR gene can lead to an increased plasma LDL level, thereby increasing the possibility of atherosclerosis and CAD [23]. Jha et al. [9] reported that the LDLR rs688 TT genotype and T allele were linked to a higher susceptibility to CAD in the Indian population. Similarly, Buraczynska et al. [14] demonstrated a strong association between the T allele and TT genotype and the presence of CVD, with significantly higher frequencies observed in the CVD‐positive subgroup. Furthermore, Martinelli et al. [8] also found that the T allele was associated with an increased risk of CAD. Lastly, Osman et al. [24] found that LDLR rs688 CT and TT genotypes were associated with an increased risk of CVD. Consistent with these reports, our study likewise revealed that the TT genotype is associated with increased susceptibility to CAD in the Bangladeshi population. This hospital‐based case–control study should be considered exploratory in nature and provides preliminary evidence for an association between LDLR rs688 polymorphism and CAD risk in a Bangladeshi cohort. The modest sample size, particularly the control group, may have limited the statistical power and generalizability of the findings. Recruitment of an age‐ and comorbidity‐matched control group was challenging in a resource‐limited setting such as Bangladesh, which resulted in a relatively small control cohort. The genotype distribution in the control group also deviated from HWE. However, genotyping accuracy was verified by repeating PCR amplification and sequencing 10% of randomly selected samples, suggesting that the deviation is more likely attributable to the small control sample size, possible population stratification, and hospital‐based sampling rather than technical error. Therefore, the findings should be interpreted cautiously and validated in larger population‐based studies.

## 5. Conclusion

The results suggest that the LDLR rs688 TT genotype is linked to an increased susceptibility to CAD among individuals in the Bangladeshi population. Moreover, patients carrying CT and TT genotypes showed significantly higher serum LDL‐C levels compared to those with the CC genotype. These findings provide preliminary evidence supporting the association between the LDLR rs688 polymorphism and CAD. However, they should be interpreted with caution. Further studies with larger sample sizes are warranted to validate these observations.

## Funding

This research did not receive any funding.

## Conflicts of Interest

The authors declare no conflicts of interest.

## Data Availability

The data supporting the findings of this study can be obtained from the corresponding author upon reasonable request.
